# Royal National Hospital for Consumption, Ventnor

**Published:** 1892-05-21

**Authors:** 


### The Institutional Workshop.

### WITHIN THE HOSPITALS.

### II.—ROYAL NATIONAL HOSPITAL FOR CONSUMPTION, VENTNOR.

The Royal National Hospital for Consumption is constructed on the separate system. That is to say, each patient is supplied with a separate bedroom. It consists at present of ten blocks, and the site provides accommodation for two others. These blocks consist of a series of houses surrounded by gardens, through which the patients are distributed. The whole are well sheltered from unfavourable winds. Each house contains separate bedrooms and sitting-rooms. The whole of the blocks are connected by a corridor in the basement, and block 9 contains a fine dining-hall where all patients who are able, partake of their meals. This dining-hall is also used as a recreation building; and it contains an organ, and here private theatricals and other entertainments are frequently given by the staff, aided by the local residents who take a deep interest in the institution. The whole of the buildings are heated by steam radiators from a central boiler house, the vitiated air being extracted by means of a powerful fan. The method employed is so powerful that it is capable of raising the temperature to upwards of 70 deg., it having been 68 deg. on the day of our inspection. It is, of course, desirable not to exceed 62 deg., a fact recognised by the medical staff who have taken steps to secure the adequate regulation of the apparatus with this object.

#### The Buildings.

The various blocks are of different sizes, blocks 1 to 8 being identical. They consist of four sitting-rooms for three patients on the ground floor, with a nurses' room, larder and box-room at the back and lavatory accommodation. The patients' bedrooms are situated upstairs in front of the building—six rooms with one bed each; the nurses and servants bed-rooms being placed at the back. Block 9 is much more extensive than the others and contains the dining-hall, which occupies the central portion of the building on the basement and ground-floors. The ground-floor also contains resident and medical officers' and chaplain's mess-room, nurses and Matron's mess-room, medical officers' consulting-room, waiting-room, telephone-room, and dispensary, lavatories, lifts, and entrance-hall. A balcony leads from the main corridor into the dining-hall. Upstairs are situated the committee-room, clinical laboratory, and accommodation for six patients' bed-rooms, together with officers and servants' quarters. Block 10 resembles the first eight blocks, except that there is a bath-room on both floors, and a ward kitchen. On the south side of each block of buildings a verandah is constructed to permit three persons to walk abreast. It is surmounted by a balcony to which the patients have access from their bed-rooms in fine weather, should they be too weak to descend. These verandahs fulfil a useful purpose by protecting the houses from heat in the summer and cold in the winter.

#### Site and Grounds.

The site consists of 22 acres, is beautifully situated and commands an uninterupted view of land and sea. It occupies one of the best parts of the under cliff at Ventnor, a well-sheltered and lovely spot, which makes an excellent site for a consumption hospital. Indeed, the grounds are so well protected from the wind that patients are nearly always able to take outdoor exercise in the grounds, which have been tastefully laid out under the superintendence of the late Sir Lawrence Peel, former Chairman of the institution. Myrtle palms, olives, and other trees thrive well in this climate, and at the present time the winter garden is bright with wild hyacinths and primroses. Altogether there are few places which should prove more beneficial to consumptives than the grounds of the Royal National Hospital.

#### The Chapel.

A handsome chapel, with accommodation for some two hundred people, has been built in the style of the 14th century. It contains many beautiful windows which have been given as memorials. The building is quite finished with the exception of a spire and south cloister, for which plans have been prepared, and both of which it is believed, will shortly be erected at the expense of a few benevolent persons. The chapel was built from funds especially subscribed for this purpose, and without any cost to the hospital. Patients are at liberty to attend any other place of worship in the neighbourhood of their own denomination, and an inmate may secure the attendance of his own minister by expressing a wish to this effect to the chaplain on his admission.

#### An Example to Other Institutions.

We have always been of opinion that it is desirable to secure the utmost personal interest in the medical institutions of this country. The soundness of this view is confirmed by the experience of the Royal National Hospital. The founders, instead of raising a large building fund, determined to bring the objects of their scheme to the notice of wealthy and benevolent people who were known to take an interest in hospitals. So successful has the new departure proved that the whole of the blocks have been erected by private friends, and each house bears a distinct name, usually that of the donor or of some relative whose name is associated therewith in memoriam. Glancing down the list we notice especially the "Charles Stuart" Hospital, a block erected in memoriam by his brother officers of the Oriental Bank Corporation. There are many hundreds of great banking and insurance corporations in this country, each and all of which should be intimately identified with the work of one or more of our great hospitals, in the same manner as the Oriental Bank. That they are not so identified at present proves that this idea has never been brought forcibly to their notice, rather than that no opportunity for a memorial has presented itself in their history. It is very often much more difficult to find a suitable object with which to commemorate the memory of a dear friend or colleague, than to raise a considerable sum for the purpose of perpetuating it. Here, then, is new and fruit ful ground which we hope both the institutions and the public will make haste to cultivate.

#### The System of Administration.

The 22nd report states, as the principal event of the year 1890, the re-organisation of the administrative staff of the hospital, rendered necessary by its recent enlargement. In the result the administration was placed in charge of a House Governor, Matron, and principal medical officer. No one familiar with the management of large institutions can fail to realise the difficulties of a Committee sitting in London, which has to control a hospital at Ventnor. The only system which could succeed in securing efficiency is one which places the entire administration in the hands of a capable Superintendent resident within the buildings. It would be unfair to him and to the committee not to make all the other resident officers subordinate, so as to enable the managers to hold the Superintendent responsible for every department, with the exception, of course, of the medical treatment of the patients. We gathered from our inspection that something is yet wanted in this direction at Ventor, and we hope that the Committee will take advantage of a change in the personnel of the staff to re-organise their system so as to fulfil the conditions we here insist upon. If this is done, then there will no doubt be the highest efficiency in every department, whereas without such a system it must always be impossible for a London Committee to secure sound management, strict economy, and perfect administration throughout the establishment.

Funds, and the Admission of Patients.

The Committee are most anxious to secure a larger subscription list, and we commend the institution to the whole country, seeing that patients are sent from all parts of the United Kingdom. There seems to be but little difficulty in securing wealthy donors to build memorial blocks, but there is a great need for many more benevolent people to become annual subscribers to this national charity. Many persons are fond of saying that the hospitals give too much free medical relief, and all such critics have here an opportunity of supporting their opinions by contributing to the Royal National Hospital. Although no person is eligible for admission unless he be necessitous, and not in a position to defray the entire cost of maintenance and medical treatment, each patient is required to pay 10s. per week, and to deposit a guarantee fee of £1 on entrance. The total amount received from patients payments in 1890 was £2,812 towards a total expenditure of upwards of £11,000. The annual subscriptions amount to less than £2,000 a year, and we do most earnestly commend these facts to the grave consideration of every family which has suffered from the scourge of consumption, in the hope that the subscription list may be promptly raised to £5,000 per annum. To what better object can any person devote a few guineas per annum?

